# Toward healthy air quality under CMIP6 scenarios: insights into the compliance with the new WHO guidelines in China

**DOI:** 10.3389/fpubh.2026.1803304

**Published:** 2026-03-16

**Authors:** Ziqi Jia, Yue Yuan, Xurong Wang, Fuzhen Shen

**Affiliations:** 1 China Industrial Culture Research Center, Nanjing University of Science and Technology, Nanjing, Jiangsu, China; 2 Key Laboratory for Meteorological Disaster Prevention and Mitigation of Shandong, Jinan, Shandong, China; 3 Jining Meteorological Bureau, Jining, China; 4 Institute of Climate and Energy Systems-Troposphere (ICE-3), Forschungszentrum Jülich GmbH, Jülich, Germany; 5 Institute of Climate and Energy Systems-Stratosphere (ICE-4), Forschungszentrum Jülich GmbH, Jülich, Germany

**Keywords:** air quality compliance, Health-based Air Quality Index, representative concentration pathways, WHO Guidelines, Yangtze River Delta

## Abstract

Ambient PM_2.5_ and O_3_ pollution remain major threats to public health, especially under the more stringent 2021 World Health Organization Air Quality Guidelines (WHO AQGs). We develop an integrated framework that combines bias-corrected CMIP6 simulations of PM_2.5_ and O_3_ during 2015–2100 under three representative concentration pathways (SSP-RCPs) with the Air Quality Index (AQI) and the Health-based Air Quality Index (HAQI). Using the WHO guideline thresholds and exposure-response functions, we quantify the compliance of two air pollutants and AQI/HAQI in national, regional scales, and also 25 cities in the Yangtze River Delta (YRD), China. Under SSP-RCP1–2.6, national mean PM_2.5_ concentrations declines from 17.4 to 9.0 μg/m^3^ and meet the WHO daily guideline shortly after 2020. While O_3_ decreases from 88.3 to 62.6 μg/m^3^ and remains below 100 μg/m^3^. AQI and HAQI both improve from the WHO Target-II toward Target-III after mid-century. Under SSP-RCP2-4.5, PM_2.5_ and O_3_ show only moderate improvement. AQI stays in Target-II and HAQI shifts to Target-I at the beginning. Under SSP-RCP3-7.0, O_3_ exceeds 100 μg/m^3^ after 2060s and HAQI surpasses 150 in some regions by 2090s. The YRD emerges as the long-term hotspot. In YRD, about 58% of cities achieve HAQI ≤ 50 under SSP-RCP1-2.6 after 2050s, while no city achieve this goal under the other two scenarios. Those results highlight that HAQI reveal multipollutant health burden rather than AQI and ambitious, region-specific, multipollutant control and climate-mitigation policies are essential to meet WHO health-based air quality targets.

## 1 Introduction

Ambient air pollution has a substantial adverse impact on human health (1, 2), the global ecosystems (3–5), and the Earth's climate system (6, 7). Combustion of fossil fuels, a major anthropogenic source of air pollutants, is also a primary source of greenhouse gases (8). There is also substantial epidemiological and toxicological evidence associating particulate matters and gaseous pollutants [e.g., Ozone (O_3_)] with cardiovascular disease, lung cancer, respiratory infections, and premature mortality (9–11), with fine particular matter (PM_2.5_) and O_3_ ranked as the major contributors (12). According to the World Health Organization (WHO) estimates, nearly the entire global population (approximately 99%) is exposed to air pollutant concentrations that exceed the recommended air quality guideline levels (AQG) (13). Therefore, to protect the public's health, urgent action is needed to design a dual-benefit strategies that simultaneously reduce disease burden attributable to air pollution and contribute to near- and long-term climate change mitigation.

The WHO began addressing the negative health impacts of air pollution as early as 1958. Based on scientific progress made in subsequent decades, the WHO Regional Office for Europe published the first health-based Air Quality Guidelines (AQGs) in 1987, with several updates issued since then. For example, the 2005 AQGs set guideline values for various pollutants, including PM_2.5_ and O_3_. And it also proposed interim targets to be used as benchmarks for phased management. Between 2005 and 2021, accumulating evidence demonstrated that adverse health effects occur even at low concentrations of various air pollutants (14). Accordingly, the WHO issued revised AQGs in 2021 with even lower recommended limit values for six air pollutants: PM_2.5_, O_3_, coarse particular matters (PM_10_), nitrogen dioxide (NO_2_), carbon monoxide (CO), and sulfur dioxide (SO_2_). Compared with the 2005 WHO AQGs, the 2021 update substantially tightens the recommended levels. For PM_2.5_, the annual guideline is reduced from 10 to 5 μg/m^3^ and the 24-h guideline from 25 to 15 μg/m^3^. For O_3_, The 2021 guidelines introduced a new peak season (6-month average) metric of to address the long-term health effects. But the short-term exposure guideline reminds that the annual 99th percentile of the daily maximum 8-h average surface O_3_ concentration (DMA8-O_3_) should not exceed 100 μg/m^3^. Overall, the 2021 WHO AQGs substantially tightened guideline levels for the six pollutants. It emphases the need to shift from “meeting concentration standards” to “meeting health standards (15).”

To protect the public health in China, the central government has promulgated the Ambient Air Quality Standards [AAQS: (GB 3095-2012, revised in 2018)] and the Technical Regulation on the Ambient Air Quality Index (AQI; HJ 633-2012). To this end, successive clean-air actions have been implemented since 2013 (16). In particular, the Action Plan for Air Pollution Prevention and Control and the Three-Year Action Plan for Winning the Blue-Sky Defense Battle are with focus on the Jing-Jin-Ji (JJJ) region, the Yangtze River Delta (YRD) and the Pearl River Delta (PRD) (17–20). These measures have driven substantial reductions in annual mean PM_2.5_ concentrations and significant increase in annual mean O_3_ concentration (10). From 2013 to 2020, the national population-weighted mean PM_2.5_ concentration in China decreased from 61.8 to 32.1 μg/m^3^ (95% CI: 26.9–37.3 μg/m^3^), corresponding to a reduction of nearly 30 μg/m^3^. However, it should be noted that this level still exceeds the 2021 WHO annual guideline of 5 μg/m^3^ by more than a factor of six (21). Existing research on air quality in China has mainly examined the current AQI system based on the AAQS, highlighting the spatiotemporal patterns of air quality in different urban clusters (UCs) and the role of meteorological modulation (16). However, the AQI framework based on a single pollutant cannot adequately characterize the combined health effects arising from simultaneous exposure to multiple pollutants (22). To overcome this limitation, the Aggregate AQI (AAQI), the Air Quality Health Index (AQHI), and the Health-based Air Quality Index (HAQI) have been developed and applied in practice (2, 23, 24). For instance, the HAQI proposed by Hu et al. (22) integrates the effects of multipollutant (e.g., PM_2.5_ and O_3_), thereby addressing the limitation of the AQI. Shen et al. (16) applied the HAQI to 367 cities across China to explore the socioeconomic factors influencing HAQI changes. Zhou et al. (25) constructed the HAQI for 366 Chinese cities and demonstrated that organic aerosols predominantly drive PM_2.5_-related health risks. One of our previous study expanded on these studies by investigating the number of deaths associated multipollutant exposure in different functional areas within a city (11). Here, we intentionally use HAQI rather than AQHI or AAQI. This is because AQHI is an air quality classification system originally developed in Canada. It is an hourly risk evaluation tool based on short-term excess mortality risks from a specific group of pollutants (e.g., NO_2_, O_3_, PM_2.5_, and PM_10_). It has its own scale (typically: 0–10) and categories (24). In contrast, AAQI is designed by aggregating individual AQIs without considering the exposure-response effects (2). HAQI extends the conventional AQI framework by integrating exposure-response functions for multiple pollutants (PM_2.5_ and O_3_ in this case) while preserving the familiar AQI scale. Therefore, it is suitable to be directly comparable with AQI while reflecting multi-pollutant health risk. When developing HAQI, it is assumed that the additional risks associated with PM_2.5_ and O_3_ were additive. And it did not simulate the potential interactions between pollutants. While this assumption is consistent with many previous multi-pollutant health risk assessments (16, 22, 25).

In addition to clean air policies, climate mitigation measures that reduce fossil fuel consumption also yield substantial co-benefits for air quality (26, 27). In 2020, the Chinese government also pledged to peak carbon emissions by 2030 and achieve carbon neutrality by 2060, which will further improve air quality. Previous studies have demonstrated that both air pollution control measures and climate mitigation policies can individually lead to substantial improvements in China's future air quality (28–32). Recently, a shared socioeconomic pathway-representative concentration pathway (SSP-RCP) analysis in China suggested that a low carbon transition before 2060, coupled with air pollution control policy, could reduce the national mean PM_2.5_ exposure to approximately 8.0 μg/m^3^, with around 78% of the population exposed to levels below 10 μg/m^3^ (27).

From the perspective of global transitions in air quality governance, several key gaps remain in the above studies. Firstly, with regard to benchmark adaptation, the limited analysis of compliance with the stringent 2021 WHO AQGs restricts the precise management of health air quality associated with low-level pollutant exposure. Secondly, in terms of scenario simulation, few studies have incorporated the evolution of the air quality index (e.g., AQI/HAQI) within SSP-RCP frameworks, which is in a lack of insight into co-benefit strategies for climate change mitigation and air pollution control. Thirdly, regarding regional representativeness, while the five UCs are regarded as both economic centers and pollution hotspots in China, current studies lack integrated analyses that investigate healthy air quality transmission from national to regional and even city level scales. To address these gaps, we have developed an integrated framework that combines the 2021 WHO benchmarks with multi-scenario air quality evolutions, and expands analysis from the national, regional to city-level scales, with particular focus on the YRD region. Thereby, it would provide scientific strategy for balancing economic growth, climate mitigation and air pollution control in eastern China.

## 2 Materials and methods

### 2.1 Site and data

**Figure 1:**
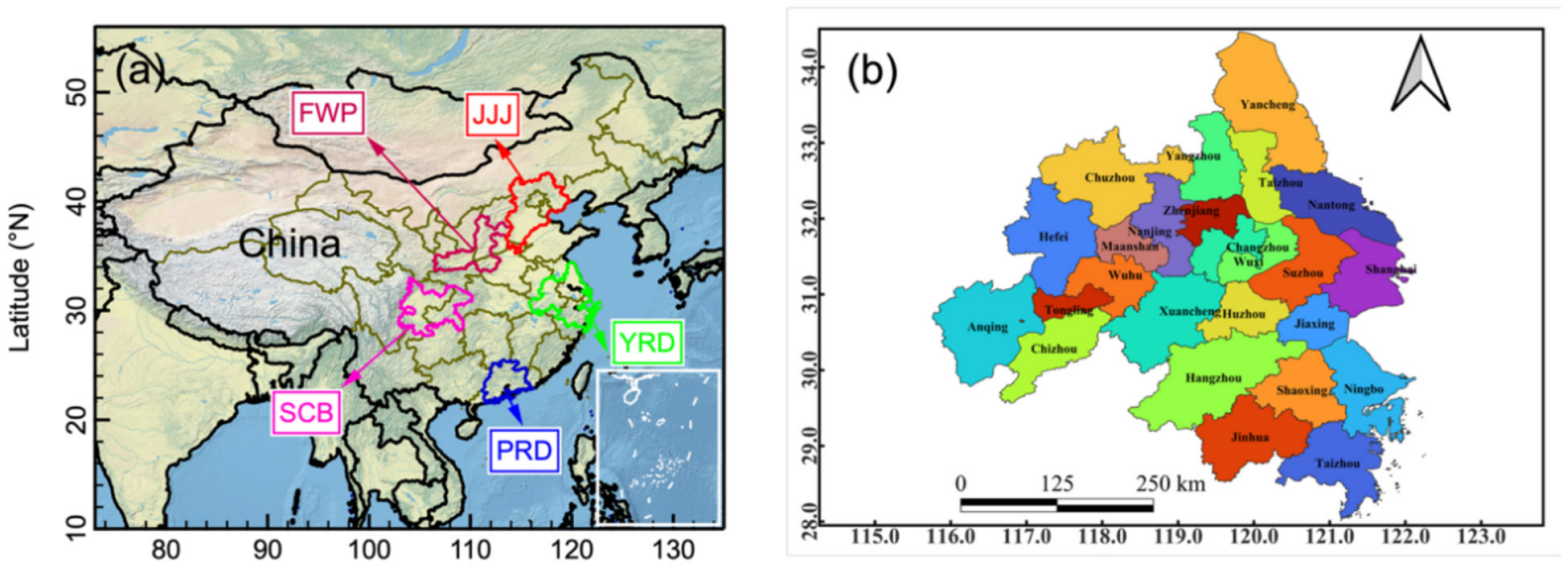
Geographical locations of five major urban clusters **(a)** and 25 cities in the YRD urban cluster **(b)** in China.

Figure 1 shows the locations of five major UCs in China (Figure 1a), including the JJJ, YRD, PRD, Sichuan Basin (SCB) and Fenwei Plain (FWP), as well as the 25 cities in the YRD region (Figure 1b). These five major UCs are the economic and industrial centers, as well as hotspots of air pollution (16). Three largest UCs are JJJ, YRD, and PRD in China, which include 53 cities in total. Despite accounting for only 3.61% of national territory, 11.9% of national cities, and 15.2% of the national population, these three UCs contributed 34.7% of China's total GDP (33). YRD cluster encompasses Shanghai city and parts of Jiangsu, Zhejiang and Anhui provinces, covering an area of roughly 2.12 × 10^5^ km^2^ and contributing approximately 25% of total GDP in China (34).

### 2.2 SSP-RCP scenario dataset

The coupled Shared Socioeconomic Pathways (SSPs) with Representative Concentration Pathways (RCPs) dataset is generated by integrated assessment models (IAMs) (35). The future emission scenarios adopt global anthropogenic and biomass burning emissions as specified in the Coupled Model Intercomparison Project Phase 6 (CMIP6) inventory. SSP1 describes a sustainable development pathway with robust economic growth, reduced inequality, behavioral change and efficiency improvements. It leads to lower emissions and strengthened environmental controls. SSP2 represents a “middle-of-the-road” trajectory. It is characterized by moderate population growth and slower convergence of incomes and energy systems that remain largely fossil-fuel-based but with gradually improving controls. SSP3 is marked by intensified regional rivalry, greater inequality, slow GDP growth concentrated in high-income countries. Whilst the renewed reliance on coal and weaker policy implementation are resulting in higher emissions of pollutants and aerosols (36). Here, three paired scenarios: SSP-RCP1-2.6, SSP-RCP2-4.5 and SSP-RCP3-7.0 were examined, which span increasing radiative forcing levels of 2.6, 4.5,and 7.0 W/m^2^ by 2100 and correspond to progressively climate mitigation challenges. SSP-RCP1-2.6 targets limiting global warming to below 2 °C relative to pre-industrial conditions; SSP-RCP2-4.5 represents an intermediate development pathway; and SSP-RCP3-7.0 constitutes a high-emission trajectory with the largest releases of CO_2_, CH_4_, and aerosol and ozone precursors. The hourly PM_2.5_ and O_3_ concentrations for 2015–2100 under each SSP-RCP scenario are simulated using the Community Earth System Model (Version 2)-Whole Atmosphere Community Climate Model Version 6 (CESM2-WACCM6), a global chemistry-climate model with a horizontal resolution of 0.90° latitude × 1.25° longitude (37). The future emissions for SSP-RCP1-2.6, SSP-RCP2-4.5, and SSP-RCP3-7.0 follow the Scenario Model Intercomparison Project SSP inventories described by O'Neill et al. (36) and Riahi et al. (38), as summarized in Turnock et al. (39). For chemistry and aerosols, CESM2-WACCM includes fully coupled troposphere-stratosphere gas-phase chemistry and a modal aerosol scheme; the chemical mechanism and aerosol treatment are now referenced explicitly to Emmons et al. (40) and Tilmes et al. (41), where the full reaction set and aerosol microphysics are documented.

### 2.3 Bias correction of the SSP-RCP dataset

Before using the model outputs of CESM2-WACCM, daily PM_2.5_ and O_3_ comparisons against the Copernicus Atmosphere Monitoring Service reanalysis dataset (CAMS) from 2003 to 2014 were made (Supplementary Figure S1). The CAMS reanalysis dataset is produced by the European Center for Medium-Range Weather Forecasts (ECMWF; https://atmosphere.copernicus.eu/) (10). From Supplementary Figure S1, it was found that WACCM-O_3_ has an overestimation significantly and WACCM-PM_2.5_ underestimates CAMS reanalysis, especially for the PM_2.5_ spikes. Therefore, different bias correction strategies are adopt to fix those biases for PM_2.5_ and O_3_. For O_3_, WACCM shows a relatively uniform positive bias with small Normalized Mean Error (NME) and high correlation, and the daily anomalies match CAMS well. Therefore, a simple additive mean-bias correction (subtracting the annual mean bias at each grid cell) is sufficient and preserves the temporal structure. For PM_2.5_, WACCM substantially underestimates high concentration episodes and shows a distribution that is too narrow relative to CAMS. The Stochastic Quantile Mapping (SQM) is thus used to correct the full distribution, especially the upper tail, while retaining day-to-day ranking. The SQM method is stochastic bias-correction or downscaling schemes designed to fix not just the distribution but also the realistic variability at local scale for the simulated climate outputs. It avoids the overestimation or underestimation problem of deterministic variance correction and quantile mapping (15, 42). After bias correction, WACCM model outputs now are agree with CAMS reanalysis well (Supplementary Figure S1). Then, we regard the baseline bias for PM_2.5_ and O_3_ as reference and apply them to three SSP-RCP scenarios. It should be noted that daily mean O_3_ here refer to the MDA8 O_3_ afterwards. The calibrated fields provide PM_2.5_ and O_3_ concentrations for cities in China from 2015 to 2100.

### 2.4 AQI and HAQI calculation


$$\begin{array}{l} AQI_{i}=\frac{AQI_{i,j}-AQI_{i,j-1}}{\left(m_{i,j}-m_{i,j-1}\right)}\times \left(m_{i}-m_{i,j-1}\right)\\ \;+AQI_{i,j-1},\;j>1 \end{array}$$ (1)

$$\begin{array}{l} AQI_{i}=AQI_{i,1}\frac{m_{i}}{m_{i,1}},\;j=1 \end{array}$$ (2)

$$\begin{array}{l} AQI=max\left(AQI_{1},AQI_{2}\cdots \;,AQI_{n}\right),\;n=1,\;2,\;\cdots \;,\;6 \end{array}$$ (3)

Equations 1–3 define the AQI, where i represents the pollutant i (PM_2.5_ and O_3_ included); *m*_*i*_ is the measured concentration of i; j is the health category index; *m*_*i, j*_is the WHO AQGs 2021 recommended interim target concentration for pollution i corresponding to the jth interim target (Table 1). Within this global target AQI system, air quality is classified into four classes according to AQI value ranges: Target III ( ≤ 50); Target-II (51–100); Target I (101–150); Over Target (≥151). In this study we adopt the 2021 WHO daily guideline values rather than the annual mean values as the breakpoint. Because both AQI and HAQI are defined on a daily basis and are used to manage short-term air quality episodes.

**Table 1:** The new WHO guidelines for short-term interim target of two air pollutants.

**WHO category**	**AQI/HAQI**	**Definitions**			
		**PM**_2.5_*	**O**_3_* **(MDA8)**	**Color**	**Air quality conditions**
		0	0		
Target-III	0–50	15	100	Green	Good
Target-II	51–100	50	120	Light-green	Health
Target-I	101–150	75	160	Orange	Unhealth
Over-Target	151–200	75^+^	160^+^	Red	Hazardous

^*^The unit of mass concentration is μg/m^3^ and the + represents the mass concentration is over the corresponding value.

The HAQI is evaluated based on the excess risk (ER) and the AQI calculation. The relative risk (RR) of air pollutants could be expressed by an exponential linear function (Equation 4). The HAQI is designed as an index establishing a threshold concentration for air pollutants, below which health risks associated with air pollution are assumed negligible. Accordingly, excess risk (ER) of mortality occurs only when pollutant concentrations exceed this defined threshold (Equation 5). Given that the ER calculation is inherently dependent on the threshold concentration (*m*_*i*, 0_), the upper limits recommended by the WHO guidelines are employed to evaluate the ERs and HAQI for six air pollutants.


$$\begin{array}{l} RR_{i}=exp\left[\beta_{i}\left(m_{i}-m_{i,0}\right)\right],\;m>m_{i,0} \end{array}$$ (4)

$$\begin{array}{l} ER_{i}=RR_{i}-1 \end{array}$$ (5)

$$\begin{array}{l} ER_{total}=\overset{n}{\underset{i=1}{\sum }}ER_{i}=\overset{n}{\underset{i=1}{\sum }}\left(RR_{i}-1\right) \end{array}$$ (6)

$$\begin{array}{l} {RR_{i}}^{*}=ER_{total}+1=exp\left[\beta \left({m_{i}}^{*}-m_{0}\right)\right] \end{array}$$ (7)

$$\begin{array}{l} {m_{i}}^{*}=ln\frac{\left(RR^{*}\right)}{\beta_{i}+m_{i,0}\;} \end{array}$$ (8)

In Equation 4, *RR*_*i*_ represents the relative risk associated with pollutant *i*, while β_*i*_ denotes the exposure-response coefficient for pollutant *i*, indicating the additional risk of mortality attributed to an incremental increase in the pollutant's concentration per unit. *m*_*i*, 0_ refers to the threshold concentration of pollutant *i*. Based on studies of short-term exposure to air pollutants and daily mortality worldwide, the β-values recommended by the WHO are 0.065 and 0.043% per 1 μg/m^3^ increase in concentration for PM_2.5_ and O_3_, respectively (43). By summing the ERs of the two air pollutants to obtain the total ER (Equation 6), the equivalent concentration of ${m_{i}}^{*}$ can be calculated using Equations 7, 8. Subsequently, the HAQI is derived from the AQI framework as the following Equations 9–11.


$$\begin{array}{l} HAQI_{i}=\frac{HAQI_{i,j}-HAQI_{i,j-1}}{\left(m_{i,j}-m_{i,j-1}\right)}\times \left({m_{i}}^{*}-m_{i,j-1}\right)\\ \;+HAQI_{i,j-1},\;j>1, \end{array}$$ (9)

$$\begin{array}{l} HAQI_{i}=HAQI_{i,1}\frac{{m_{i}}^{*}}{m_{i,1}},\;j=1 \end{array}$$ (10)

$$\begin{array}{l} HAQI=max\left(HAQI_{1},HAQI_{2}\ldots ,HAQI_{n}\right),\\ \;n=1,\;2,\;\ldots ,\;6. \end{array}$$ (11)

## 3 Results

### 3.1 Spatiotemporal comparison of decadal PM_2.5_ under each SSP-RCP scenario

**Figure 2:**
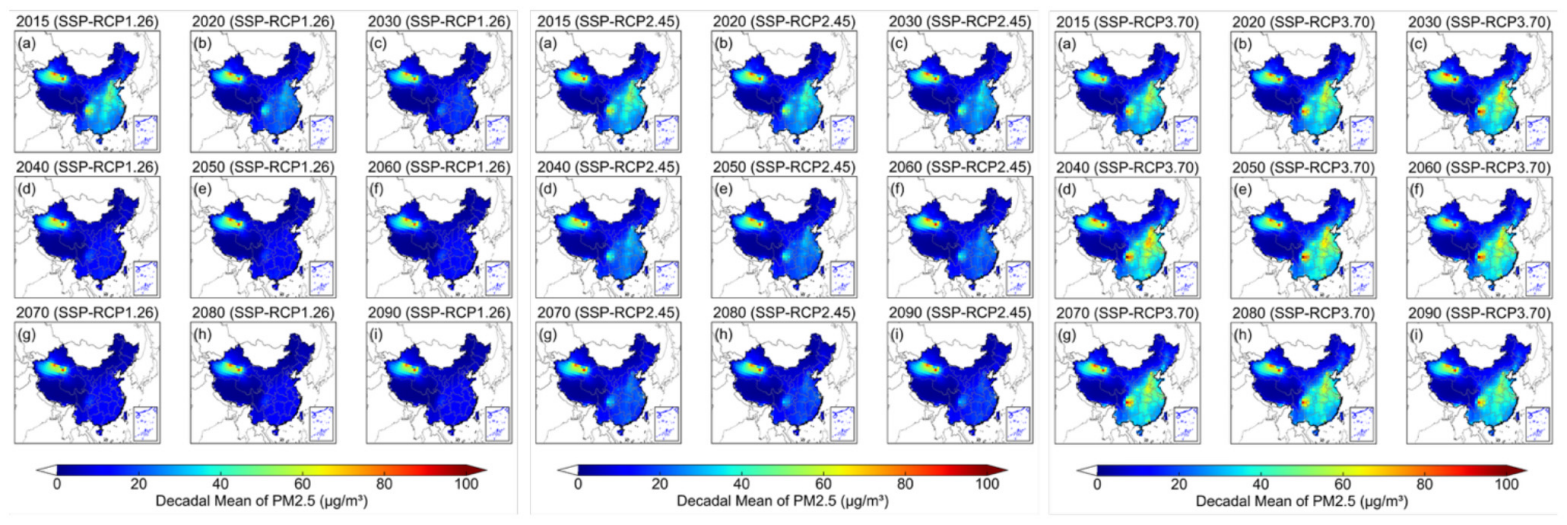
The spatiotemporal distribution of decadal mean PM2.5 from 2015 to 2090 under three different SSP-RCP scenarios (**left**: SSP-RCP1.26, **middle**: SSP-RCP2.45, **right**: SSP-RCP3.70).

Figure 2 displays the spatiotemporal distribution of decadal mean PM_2.5_ from 2015 to 2090 under the SSP-RCP1-2.6, SSP-RCP2-4.5 and SSP-RCP3-7.0 scenarios. Relative to SSP-RCP2-4.5 and SSP-RCP3-7.0, most regions of China (except for desert areas) under SSP-RCP1-2.6 maintain the lowest national PM_2.5_ levels, ranging from 9.0 to 17.4 μg/m^3^. Under this pathway, national mean PM_2.5_ in China meets the WHO AQG (daily mean) of 15 μg/m^3^ from around 2020 onward. Under SSP-RCP2-4.5, improvements in national PM_2.5_ are moderate and lie between those under SSP-RCP1-2.6 and SSP-RCP3-7.0. The decadal mean PM_2.5_ concentration decreases from 18.1 μg/m^3^ in 2015 to 11.7 μg/m^3^ in 2090, meeting the WHO AQG by the 2040s (14.5 μg/m^3^). Under SSP-RCP3-7.0, national PM_2.5_ levels deteriorate significantly until the 2060s and then begin to improve from 2070 to 2090. However, this improvement is insufficient for air quality to meet the WHO AQG across China by the end of the century, with a decadal mean PM_2.5_ level at 20.2 μg/m^3^ in 2090s. In terms of regional differences (Supplementary Figure S2), PM_2.5_ pollution is more severe in the YRD (14.1 μg/m^3^) and SCB (13.9 μg/m^3^) under SSP-RCP1-2.6. These two regions lag by about one decade in complying with the WHO AQG, reaching it in the 2040s. Whereas the JJJ (14.0 μg/m^3^), FWP (14.2 μg/m^3^) and PRD (15.0 μg/m^3^) meet the target in the 2030s. Thereafter, residents in all five urban clusters are projected to breathe air that meets the WHO daily PM_2.5_ AQG. Under SSP-RCP2-4.5, the YRD remains the most polluted cluster among the five, experiencing the highest PM_2.5_ level by the end of the 21st century (17.9 μg/m^3^), followed by the SCB (17.1 μg/m^3^) and PRD (16.8 μg/m^3^). Only the JJJ (13.8 μg/m^3^) and FWP (13.6 μg/m^3^) are projected to meet the WHO daily PM_2.5_ AQG of 15 μg/m^3^ in 2090s. Under SSP-RCP3-7.0, all five regions experience severe PM_2.5_ pollution by the end of the 21st century, with 2090 decadal mean concentrations ranging from 40.8 μg/m^3^ in the SCB, 37.5 μg/m^3^ in the YRD, 35.3 μg/m^3^ in the JJJ and 34.3 μg/m^3^ in the FWP to 30.3 μg/m^3^ in the PRD.

### 3.2 Spatiotemporal comparison of decadal O_3_ under each SSP-RCP scenario

**Figure 3:**
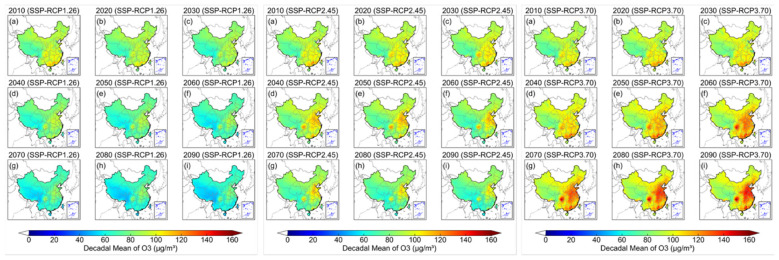
The spatiotemporal distribution of decadal mean O3 from 2015 to 2090 under three different SSP-RCP scenarios (**left**: SSP-RCP1.26, **middle**: SSP-RCP2.45, **right**: SSP-RCP3.70).

Figure 3 presents the spatiotemporal evolution of decadal mean surface O_3_ concentrations from 2015 to 2090 under the low (SSP-RCP1-2.6), medium (SSP-RCP2-4.5) and high emission (SSP-RCP3-7.0) scenarios. Compared with medium and high emission scenarios, populations across China under low emission scenarios are exposed to the lowest national O_3_ burdens, with decadal mean concentrations declining from 88.3 μg/m^3^ in 2015 to 62.6 μg/m^3^ in 2090. Under this scenario, national mean O_3_ remains consistently below the WHO AQG value of 100 μg/m3 throughout the study period. Under the medium scenario, the decadal mean O_3_ levels are lower than those under high emission scenario but higher than under low emission one. The medium scenario changes in decadal mean O_3_ across China go toward in two opposite directions. In detail, it goes up to the peak from 2015 (88.3 μg/m^3^) to 2030 (91.5 μg/m^3^) and then decreases from 2040 (91.2 μg/m^3^) to 2090 (78.9 μg/m^3^). Although the O_3_ peaks occurs around 2030s, the national mean concentration remains below the WHO guidelines value of 100 μg/m^3^. In contrast, under high emission scenario, national mean O_3_ level deteriorates substantially, with concentrations rising from 88.3 μg/m^3^ in 2015 to 104.1 μg/m^3^ in 2090s and exceeding the WHO guideline from the 2060s onward. It indicates a considerable potential threat from future O_3_ pollution.

Regionally (Supplementary Figure S3), in 2015 under low emission scenario, O_3_ pollution is most pronounced in the PRD (101.6 μg/m^3^), followed by the YRD (96.6 μg/m^3^), SCB (96.1 μg/m^3^), FWP (95.6 μg/m^3^), and JJJ (89.4 μg/m^3^), respectively. However, along this pathway, O_3_ pollution in the YRD is projected to surpass that in the other four regions over the study period, while still remaining below the WHO guideline value. Under the medium emission scenario, the five regions exhibit similar temporal change patterns in O_3_ levels, with concentrations increasing initially and then declining. The primary differences lie in the timing and magnitude of the peak concentrations. In the SCB, O_3_ levels show a peak in 2030s at 99.2 μg/m^3^, with concentrations in all other decades remaining below the WHO guideline. In the PRD, the O_3_ peak occurs in the same decade but reaches 105.6 μg/m^3^, exceeding the WHO guideline. In contrast, the FWP city cluster lags by roughly two decades, with O_3_ peaking at 100.3 μg/m^3^ in 2050s. The YRD and JJJ both reach their O_3_ peaks one decade later, with decadal mean concentrations of 107.2 and 107.7 μg/m^3^ in 2040s, respectively. However, they do not fall below the WHO guideline until 2070s (98.4 μg/m^3^) in the YRD and 2080s (99.4 μg/m^3^) in the JJJ, corresponding to delays of 3 and 4 decades relative to their peak decades. Under high emission scenario, residents in all five city clusters face progressively worsening O_3_ pollution, with decadal mean concentrations exceeding the WHO guideline. By 2090s, the YRD remains the most severe affected city cluster, with decadal mean concentrations ordered as follows: YRD (128.8 μg/m^3^), JJJ (128.4 μg/m^3^), FWP (120.8 μg/m^3^), PRD (117.3 μg/m^3^) and SCB (116.2 μg/m^3^).

It is important to emphasize that the three SSP-RCP pathways are representations of different global development and policy futures, but not forecasts for China. In practice, China's recent air quality and climate policies characterized by a significant decrease in PM_2.5_ since 2013. In China, the objective of peaking CO_2_ emissions before 2030 and achieving carbon neutrality by 2060 are most similar to a trajectory between SSP-RCP1-2.6 and SSP-RCP2-4.5. Therefore, SSP-RCP1-2.6 can be interpreted as an ambitious co-benefits pathway. SSP-RCP2-4.5 can be regarded as the moderate implementation of current pledges. SSP-RCP3-7.0 is a counterfactual scenario with high-emissions. It represents a world with weaker climate and air pollution controls. The significant deterioration in PM_2.5_ and O_3_ levels under the SSP-RCP3-7.0 scenario should therefore be considered an upper limit on potential health risks rather than a probable outcome for China.

### 3.3 Spatiotemporal comparison of decadal AQI/HAQI under each SSP-RCP scenario

**Figure 4:**
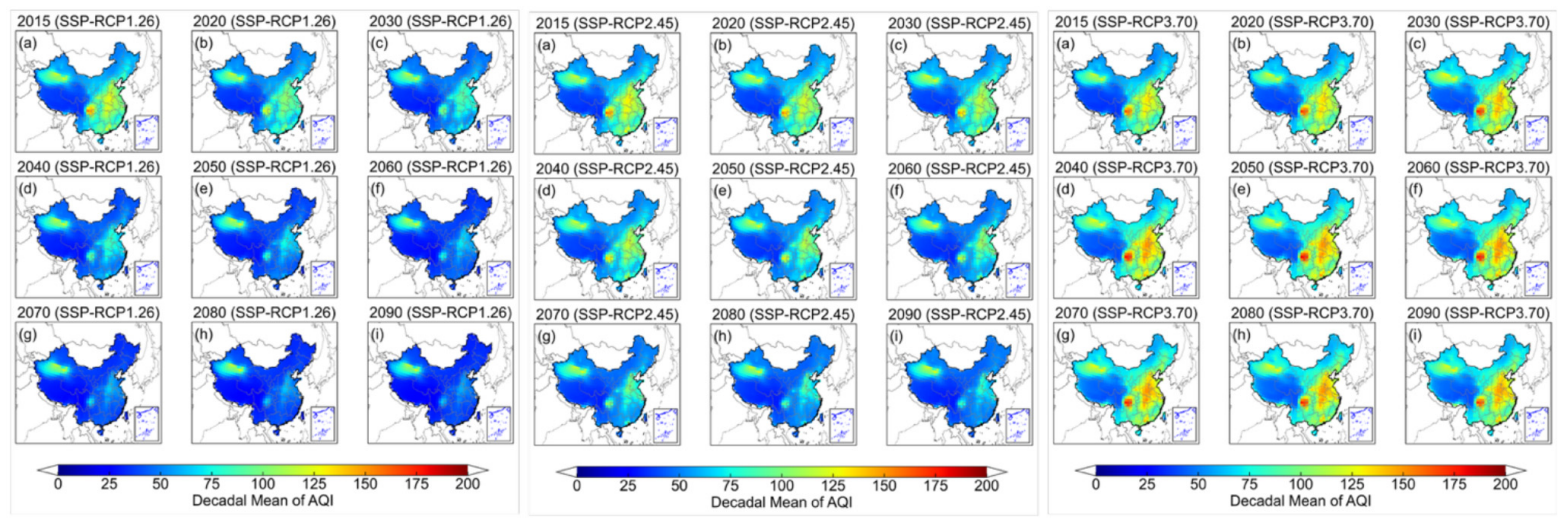
The spatiotemporal distribution of decadal mean AQI from 2015 to 2090 under three different SSP-RCP scenarios (**left**: SSP-RCP1.26, **middle**: SSP-RCP2.45, **right**: SSP-RCP3.70).

After separate analyses of the national- and regional-scale spatiotemporal distributions of PM_2.5_ and O_3_, the AQI and HAQI are computed from these two pollutants to characterize ambient air conditions and the associated health risks exposed to populations. Figure 4 illustrates the spatiotemporal distribution of decadal mean AQI from 2015 to 2090 under the ambitious- (SSP-RCP1-2.6), intermediate- (SSP-RCP2-4.5) and less ambitious emission reduction (SSP-RCP3-7.0) pathways. Compared with intermediate and less ambitious pathways, populations across China under ambitious pathway experience the lowest national AQI levels, with decadal mean values declining from 66.7 in 2015 to 39.4 in 2090s. Along this pathway, the national mean AQI lies within the WHO Target-II category during 2015–2030s (66.7–53.8) and within WHO Target-III category during 2040s−2090s (49.9–39.4). Under intermediate pathway, AQI levels are consistently higher than under ambitious pathway but lower than under less ambitious pathway, decreasing from 67.7 in 2015 to 51.1 in 2090s. However, the national mean AQI persists within the WHO Target-II category throughout the study period and does not attain the WHO guideline level. In contrast, under less ambitious pathway, the national mean air quality deteriorates steadily, with AQI increasing from 69.6 in 2015 to 81.5 in 2090s and exceeding the WHO Target-III category from 2015 onward. It indicates a growing health burden associated with degraded air quality in the future.

**Figure 5:**
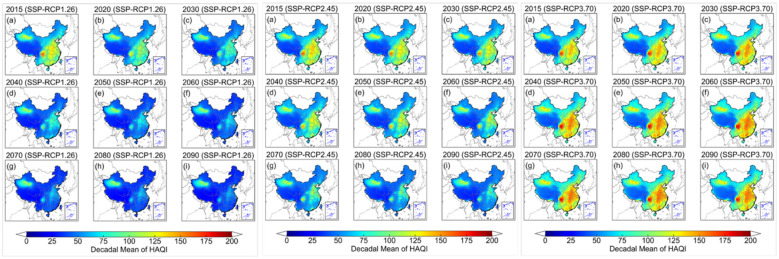
The spatiotemporal distribution of decadal mean HAQI from 2015 to 2090 under three different SSP-RCP scenarios (**left**: SSP-RCP1.26, **middle**: SSP-RCP2.45, **right**: SSP-RCP3.70).

Similar to the AQI assessment, we further calculate the HAQI to evaluate how multi-pollutant health risks vary under the same scenarios, relative to the changes captured by the single-pollutant based AQI (Figure 5). Compared with AQI, HAQI generally yields higher index values and shows slower improvements in air quality, particularly in densely populated and industrialized regions. Because it captures not only the effect of a single pollutant but the joint health burden of PM_2.5_ and O_3_ when both exceed the baseline concentrations defined by the WHO guideline values. Under SSP-RCP1-2.6, HAQI also declines over time, from 71.5 in 2015 to 39.8 in 2090. However, HAQI is projected to meet the WHO Target-III threshold by 2050s (47.2). It is lagging the AQI by one decade as AQI attains Target-III in 2040 (49.9). Under SSP-RCP2-4.5, HAQI indicates only modest improvements and remains within the WHO Target-II category. It decreases from 73.0 in 2015 to 52.4 in 2090s. Under SSP-RCP3-7.0, increases in HAQI are more pronounced than those in AQI with more widespread HAQI hotspots emerging. This situation underscores that the deterioration of combined PM_2.5_ and O_3_ exposure under this high emission pathway poses a substantially greater health threat than that from singled pollutant based AQI.

### 3.4 Regional compliance of AQI/HAQI to the new WHO AQGs

**Figure 6:**
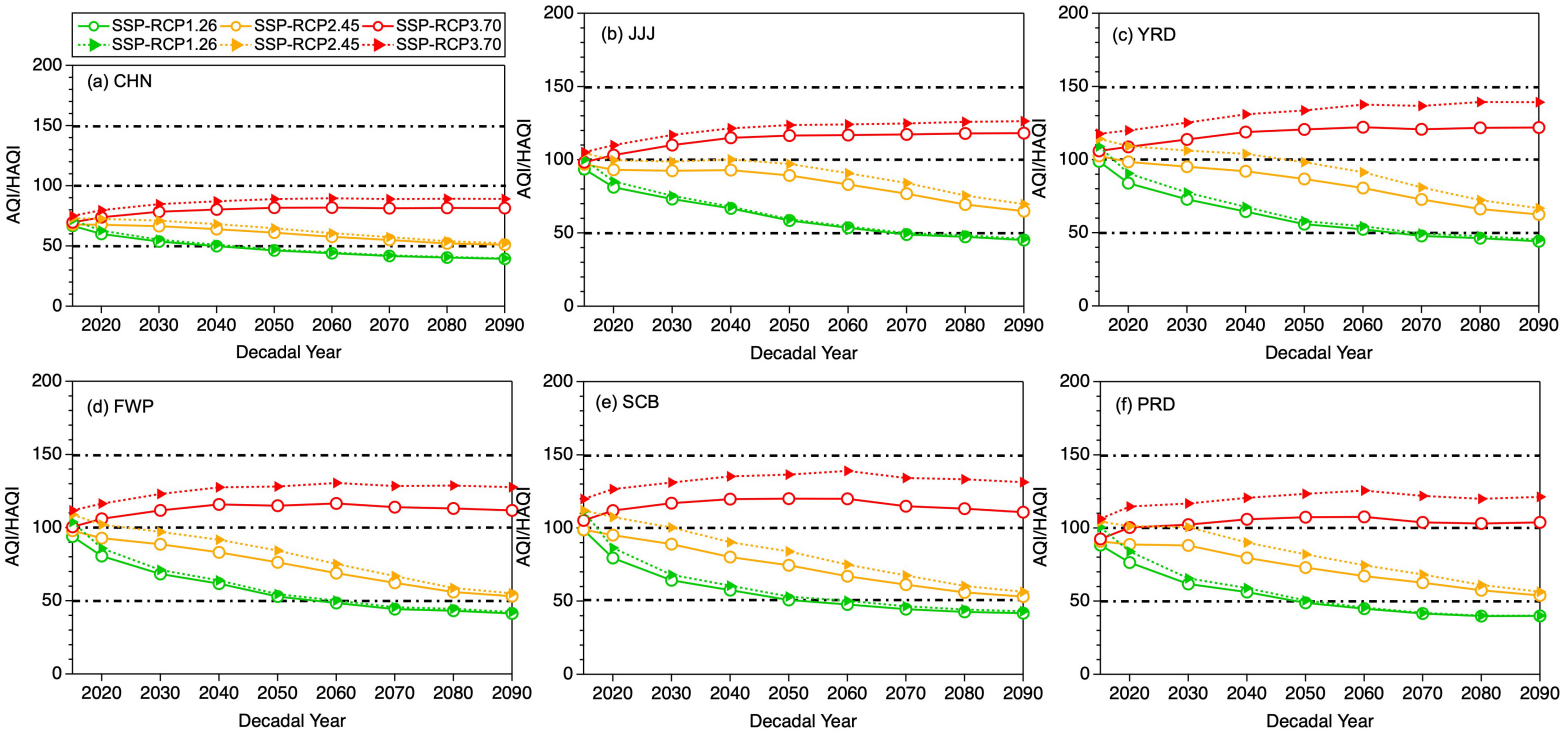
The temporal distributions of decadal mean AQI (solid marker line)/HAQI (dash-marker line) from 2015 to 2090 across China and five city clusters under three different SSP-RCP scenarios (green marker line: SSP-RCP1.26, brown marker: SSP-RCP2.45, red marker line: SSP-RCP3.70).

Figure 6 shows the time series of decadal mean AQI/HAQI across China and five UCs under the SSP-RCP1-2.6, SSP-RCP2-4.5 and SSP-RCP3-7.0 scenarios. Overall, China and the five UCs exhibit generally consistent patterns in AQI and HAQI under the three SSP-RCP scenarios. Under SSP-RCP1-2.6, AQI declines steadily in all regions, with the PRD (48.9) reaching the WHO Target-III category earliest in 2050s, followed by the FWP (48.5) and SCB (47.8) in 2060s, and the JJJ (48.9) and YRD (47.9) in 2070s. For HAQI compared to AQI, three city clusters show a one-decade delay in transitioning from the WHO Target-II to Target-III category, with a shift point at 2060s in the PRD (45.7) and 2070s in the FWP (45.6) and SCB (46.6). While the shift point in JJJ (49.9) and YRD (49.5) still remain in 2070. Under SSP-RCP2-4.5, regional AQI continues to improve but at a slower pace, staying within the WHO Target-II category. And the JJJ (65.0) and YRD (62.5) keep as the most polluted clusters throughout the period by 2090s. In contrast, HAQI also decreases but spans both the WHO Target-II and WHO Target-I categories. Particularly, the mean HAQI level (93.7) over the whole period in YRD is the highest, followed by the JJJ (91.2), SCB (83.7), FWP (82.2), and PRD (82.1), respectively. These results imply that when HAQI is applied to assess regional air quality, air quality will deteriorate in varying degrees and even step into a more polluted category. In the SSP-RCP3-7.0 scenario, both AQI and HAQI increase or remain elevated in all regions. By 2090, AQI in the YRD (122.0) ranks as the highest among the five clusters, followed by JJJ (118.2), FWP (111.8), SCB (110.8) and PRD (103.9), respectively. The rise in HAQI is more pronounced than that in AQI in the YRD (139.2) relative to the SCB (131.3), JJJ (126.4), FWP (127.6), and PRD (121.2). It highlights that under this scenario populations in the YRD face a substantial and widening health risk from multi-pollutant exposure despite only modest changes in AQI.

### 3.5 City-level compliance of AQI/HAQI to the 2021 WHO AQGs in YRD

**Figure 7:**
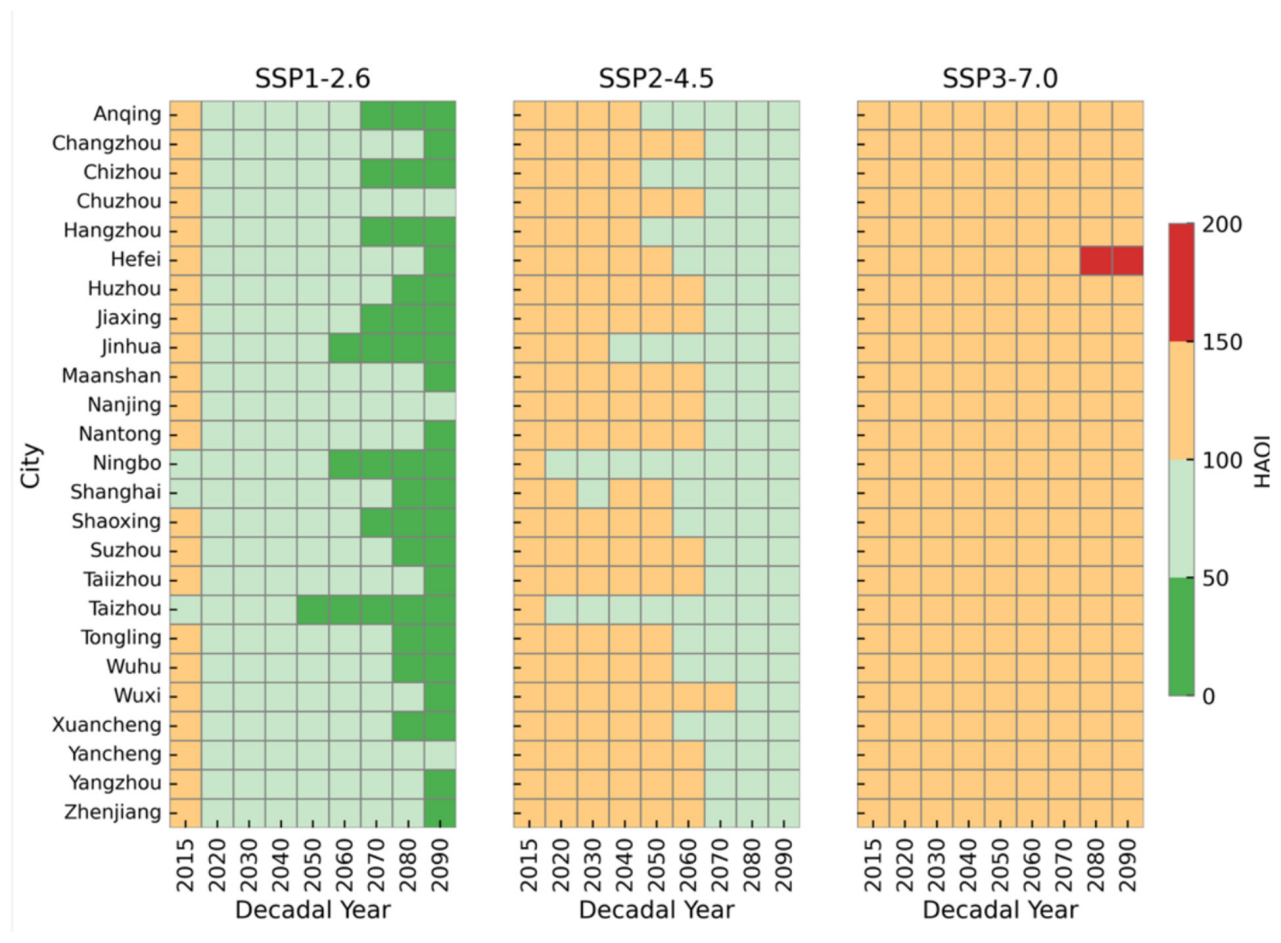
The compliance of HAQI across YRD cities under SSP-RCP1-2.6 (**left panel**), SSP-RCP2-4.5 (**middle panel**) and SSP-RCP3-7.0 (**right panel**).

Given the projected highest HAQI in the future, the city level compliance with WHO guidelines in the YRD is demonstrated in Figure 7. It shows if and when the future decadal mean HAQI values in individual cities of the YRD are projected to enter the WHO Target-III category (HAQI: 0–50) under the three SSP-RCP scenarios. As shown in the figure, almost all YRD cities lie within the WHO Target-I (HAQI: 100–150) in 2015. And the majority of them are not expected to achieve the WHO Target-III HAQI level in the near term. Only under the strongest mitigation scenario (SSP-RCP1-2.6), a large number of cities eventually turn to the green color, whereas under SSP-RCP2-4.5 and SSP-RCP3-7.0 no cities are projected to achieve HAQI ≤ 50.

Under SSP-RCP1-2.6, air quality improves steadily after 2015 in all cities, with decadal mean HAQI values shifting from the orange range (HAQI: 100–150) to the light-green range (HAQI: 50–100) by around 2040. The first clear compliance with the WHO Target-III HAQI category occurs in the 2050s in Taizhou (Zhejiang Province), where relatively strong emission reductions are projected. From the 2060s onward, more cities start to comply: coastal and economically developed cities such as Ningbo, Jinhua, Shaoxing, Hangzhou and Shanghai all meet the WHO Target-III (HAQI: 0–50) category by 2070s. By the 2080s, 58% of the 25 YRD cities-including Anqing, Chizhou, Hangzhou, Huzhou, Jiaxing, Jinhua, Ningbo, Shanghai, Shaoxing, Suzhou, Taizhou, Tongling, Wuhu, Xuancheng-have entered the WHO Target-III HAQI category. However, a group of more inland or industrial cities (Chuzhou, Nanjing and Yancheng) remain in the WHO Target-II HAQI category even at the end of the century. The evolution of HAQI under SSP-RCP2-4.5 is significantly different. Along this pathway, none of the YRD cities reach the WHO Target-III level at any time between 2015 and 2090. Instead, all cities remain confined to the WHO Target-I and WHO Target-II categories. During the first half of the century (2015–2050), most cities stay in the WHO Target-I range (HAQI: 100–150), with only shortly stay in the WHO Target-II (HAQI: 50–100) in some decades and cities. From about 2060 onwards, the majority of cities show a transition from orange to light-green, indicating that almost all YRD cities have decadal mean HAQI values below 100 by 2070–2090. Nevertheless, by the end of the century the region as a whole would still fall short of the “good” air-quality level (HAQI ≤ 50), and the overall improvements remain modest compared to SSP-RCP1-2.6. Under the SSP-RCP3-7.0 scenario, air quality deteriorates further. Throughout the entire period, all cities are covered by the orange color, with HAQI values around 100–150, indicating persistently poor air quality. No city shows any decade with HAQI below 50, resulting in zero compliance with the WHO Target-III category. Moreover, in the 2080s and 2090s one city (Hefei) even enters the WHO Over-Target (HAQI ≥ 150), implying a shift to “very unhealthy” conditions in the later century. This scenario therefore represents a future in which current high HAQI levels in the YRD not only fail to improve, but in some locations become even worse.

Overall, these results clearly show that compliance with the HAQI green category (0–50) in YRD cities is only feasible under the strong-mitigation pathway SSP-RCP1-2.6, and mainly after 2050. Under SSP-RCP2-4.5, air quality improves from WHO Target-I to WHO Target-II but never attains the WHO Target-III. Whereas under SSP-RCP3-7.0 all cities remain in poor air-quality conditions throughout the century. This comparison underscores that ambitious emission reductions are indispensable for HAQIs in YRD cities to enter and remain within the WHO Target-III category, and that weaker or delayed control efforts would leave the region far from this target for the foreseeable future.

## 4 Discussion

### 4.1 Prospects for achieving the 2021 WHO AQGs under different SSP-RCPs

Our results emphasis the critical influence of future socioeconomic development and climate-mitigation pathways on China's ability to meet the 2021 WHO AQGs. Under SSP-RCP1-2.6, both PM_2.5_ and O_3_ concentrations continue to decline nationwide, with China as a whole is projected to meet the daily PM_2.5_ guideline (15 μg/m^3^) shortly after 2020. This finding is line with a SSP-RCP study across European countries, which indicates the SSP-RCP1-2.6 is the only pathway showing PM_2.5_ decline and approaching the annual mean WHO target by 2040s (15). O_3_ remains below the short-term WHO guideline (100 μg/m^3^) throughout the 21st century. The corresponding AQI and HAQI trajectories show that, in this low-emission scenario, air quality steadily transitions from WHO Target-II levels (AQI/HAQI: 50–100) toward the WHO Target-III category (AQI/HAQI: 0–50). However, most regions in China only reach HAQI ≤ 50 in the second half of the century. These patterns demonstrate that the revised WHO guidelines could be achieved under stringent climate mitigation and air pollution controls, but only with sustained effort over several decades. By contrast, SSP-RCP2-4.5 represents a “partial-success” pathway. National mean PM_2.5_ gradually decreases and eventually meets the WHO daily guideline after around 2040s. However, O_3_ follows an “up-down” trajectory, peaking around 2030s and declining only moderately. Consequently, the AQI improves but remains confined to WHO Target-II levels by the end of the century. Meanwhile HAQI in many regions still partly lies within the WHO Target-I range (100–150). This indicates that moderate climate and air quality policies can substantially reduce PM_2.5_ related risks. They are insufficient to eliminate the combined health impacts of PM_2.5_ and O_3_ exposure. In the high-emission SSP-RCP3-7.0 pathway, both pollutants remain at elevated levels or deteriorate further. PM_2.5_ concentrations rise well above the WHO guideline by the middle of century and remain high even after improvements in the late years of the century. While O_3_ exceeds 100 μg/m^3^ nationally from the 2060s onward. Consequently, both the AQI and the HAQI increase, with the HAQI in many regions entering or approaching the Over-Target category (≥150) by 2090s. This scenario is projected to result in substantial health costs due to delayed or inadequate emission controls, as populations would be exposed to persistent and even worsening risks from multiple pollutants despite possible long-term climate feedbacks that would slightly alleviate PM_2.5_ in the very late century.

It is important to emphasize that the three SSP-RCP pathways are representations of different global development and policy futures, but not forecasts for China. In practice, China's recent air quality and climate policies characterized by a significant decrease in PM_2.5_ since 2013. In China, the objective of peaking CO_2_ emissions before 2030 and achieving carbon neutrality by 2060 are most similar to a trajectory between SSP-RCP1-2.6 and SSP-RCP2-4.5. Therefore, SSP-RCP1-2.6 can be interpreted as an ambitious co-benefits pathway. SSP-RCP2-4.5 can be regarded as the moderate implementation of current pledges. SSP-RCP3-7.0 is a counterfactual scenario with high-emissions. It represents a world with weaker climate and air pollution controls. The significant deterioration in PM_2.5_ and O_3_ levels under the SSP-RCP3-7.0 scenario should therefore be considered an upper limit on potential health risks rather than a probable outcome for China.

Overall, the three SSP-RCP pathways demonstrate that coordinated control of both primary particles and precursor gases is an effective strategy for achieving the new WHO health-based guidelines. Modest emission reductions are insufficient. Strong and sustained mitigation is required to meet the WHO Target-III category for HAQI across China.

### 4.2 Implication of HAQI relative to the current AQI

By taking into account the combined health effects of multiple pollutants, the HAQI system provides information that the AQI system is not captured (2, 22). Across all scenarios, HAQI values are consistently higher than AQI, and improve more slowly. Even under SSP-RCP1-2.6, while the AQI reaches WHO Target-III levels nationally by around 2040s, the HAQI lags by approximately 10 years. Under SSP-RCP2-4.5, the AQI in many regions remains within Target-II and gives an impression of “acceptable” air quality. However, HAQI shows that the combined health risks of PM_2.5_ and O_3_ still fall within Target-I or even Over-Target categories. This difference arises because the AQI is determined by the worst pollutant on a given day, and does not account for the cumulative risk of exposure to multiple pollutants that simultaneously exceed their health-based thresholds (11). HAQI, in contrast, aggregates ER across pollutants within a relative-risk framework (16). Our results show that when PM_2.5_ concentrations approach WHO guideline values but O_3_ remains elevated. The AQI can improve slowly while the HAQI remains comparatively high. This pattern is particularly pronounced under SSP-RCP2-4.5 and SSP-RCP3-7.0 in eastern China, where controls on PM_2.5_ emissions alone cannot fully offset the increasing burden of O_3_.

These findings support the argument that future air quality management in China should transition from a concentration-based, single-pollutant AQI toward a health-based, multi-pollutant index such as HAQI. HAQI provides policymakers with a more realistic measure of compound health risks, enabling them to develop comprehensive strategies that target both particulate and O_3_ precursors, rather than focusing solely on PM_2.5_.

### 4.3 Regional differences and vulnerability of the YRD

Although national mean projections reveal significant variations among the scenarios, the health impact of air pollution varies considerably across regions (44). The five major UCs examined here consistently exceed the national mean and exhibit distinct temporal trajectories. Even under the low-emission SSP-RCP1-2.6 scenario, the YRD and SCB fall short of the JJJ, FWP, and PRD in terms of achieving the WHO PM_2.5_ guideline, and do not attain decadal mean PM_2.5_ concentrations below 15 μg/m^3^ by 2040s. For O_3_, the YRD emerges as a persistent hotspot: under SSP-RCP1-2.6, it eventually surpasses the other clusters in O_3_ levels. Under SSP-RCP3-7.0, it becomes the most polluted region by 2090s, with decadal mean O_3_ approaching 130 μg/m^3^. These regional patterns of changes in PM_2.5_ and O_3_ are reflected in AQI and even more strongly in HAQI. Under SSP-RCP2-4.5, the AQI indicates that the JJJ and YRD are only slightly worse than the other clusters by 2090s. However, the HAQI clearly identifies the YRD as having the highest multi-pollutant health risk (average HAQI: 94), followed by the JJJ and SCB. In SSP-RCP3-7.0 scenario, the YRD again stands out with both AQI and HAQI exceeding 120 and 135, respectively, indicating widespread unhealthy air quality. This regional vulnerability likely reflects the combined influence of dense populations, intensive industrial and transport emissions, complex meteorology, and regional transport of pollutants (45).

City-level analysis in the YRD further reveals significantly inequities within the region. Even under SSP-RCP1-2.6, only around 58% of YRD cities are projected to fall into the HAQI ≤ 50 category by the 2080s, while several heavily industrialized or inland cities (e.g., Chuzhou, Nanjing, Yancheng) remain in WHO Target-II. Under SSP-RCP2-4.5, no YRD city ever meets the category of HAQI ≤ 50. And under SSP-RCP3-7.0, all cities persist in Target-I or worse. By the end of the century, Hefei will have entered Over-Target levels. These findings suggest that national-level compliance statistics can mask substantial regional and urban disparities, highlighting the need for targeted interventions in high-risk cities and sectors within the YRD.

### 4.4 Policy implications and co-benefits for climate and health

Our integrated AQI/HAQI framework, which is based on the 2021 WHO AQGs and SSP-RCP scenarios, provides several policy-relevant implications: (1) alignment of climate and air quality goals. The strong climate mitigation pathway of SSP-RCP1-2.6, which is conceptually aligned with China's “carbon neutrality” pathway, yields the most substantial reductions in both PM_2.5_ and O_3_. It is also the only pathway under which large-scale HAQI compliance (HAQI ≤ 50) becomes achievable. This demonstrates that ambitious greenhouse gas mitigation, together with stringent air pollution controls, can generate significant health benefits; (2) the importance of multipollutant control. Under intermediate and high-emission pathways, isolated PM_2.5_ reductions are insufficient because O_3_ either remains high or worsens. Effective strategies must therefore jointly address primary particle emissions and the precursors of O_3_ and secondary aerosols, including NO_x_, VOCs, and SO_2_. It is also necessary to account for non-linear chemical interactions that may lead to unintended increase in O_3_ levels (45); (3) the need for region-specific policies. The pronounced regional contrasts revealed by HAQI, especially in the YRD, suggest that uniform national policies may fail to achieve equitable health outcomes. Region-specific emission-reduction strategies that consider local industrial structures, energy sources, and meteorological conditions are required. For instance, further reducing industrial and shipping emissions, replacing petrol-powered vehicles with electric ones, and controlling VOCs may be particularly effective in YRD coastal cities. Meanwhile, cleaner residential fuels and regional transport management would be the key in inland areas; and (4) Health-oriented governance metrics. As HAQI more accurately captures the health risks posed by multiple pollutants, it should be progressively incorporated into environmental assessments, early warning systems, and the performance evaluation of regional air quality plans.

### 4.5 Limitations and future research

Several limitations of this study should be acknowledged. Firstly, our projections are based on bias-corrected CESM2-WACCM6 simulations compared with the CAMS dataset from 2003 to 2014. Although this calibration improves the model's accuracy, uncertainties remain, particularly with regard to the representation of O_3_ chemistry, aerosol formation, and boundary-layer processes. Secondly, HAQI is calculated using a fixed set of WHO-recommended exposure-response coefficients (β), which primarily are derived from global multi-city time-series studies. These coefficients may not fully capture regional variations in baseline health exposure, vulnerability, or healthcare access in China, nor account for possible non-linearities at very low or very high pollution concentrations. Thirdly, our analysis focuses on area-weighted PM_2.5_ and O_3_ rather than the population weighted ones, which might underestimate or misrepresent health impacts in densely populated or rapidly urbanizing regions. Another problem is that it does not include all relevant pollutants. As a result, future work could extend the HAQI framework to include PM_10_, NO_2_, SO_2_, and CO and transfer it from area weighted index to population weighted index. Finally, our analysis uses decadal means and does not consider extreme events or chemical components interactions (e.g., heat-related O_3_ spikes or PM_2.5_-O_3_ interaction), which can drive significant short-term health impacts. Addressing these limitations will require the coupling of high-resolution regional chemistry-transport models with more accurate health-impact models, and the further integration of local observational networks and epidemiological data. Nevertheless, this study represents a significant step in assessing HAQI under various socioeconomic and climate scenarios.

## 5 Conclusion and remarks

Based on bias-corrected CMIP6 simulations of hourly PM_2.5_ and O_3_ from 2015 to 2100 under three SSP-RCP scenarios, the compliance of two individual pollutants (PM_2.5_ and O_3_) and two air quality indexes (AQI and HAQI) with the WHO AQGs for 2021 was investigated at national, regional and city-level scales. Firstly, only the strong mitigation pathway can achieve WHO guideline levels on a large scale. Under SSP-RCP1-2.6, the national mean of PM_2.5_ falls from approximately 17.4 to 9.0 μg/m^3^, while the national mean of O_3_ falls from 88.3 to 62.6 μg/m^3^. This keeps O_3_ below 100 μg/m3 throughout and brings PM_2.5_ below the daily WHO guideline shortly after 2020. The AQI and HAQI both decline from around 66.7 to 39.4, with the AQI entering the WHO Target III category in the 2040s and the HAQI in the 2050s. Under SSP-RCP2-4.5, PM_2.5_ improves, but O_3_ declines only modestly. By the 2090s, the AQI remains in the Target II category (approximately 50), and the HAQI spans the Target II/Target I category. The worst outcome is produced by SSP-RCP3-7.0: national mean O_3_ exceeds 100 μg/m^3^ after the 2060s, with HAQI rising above 150 in some regions by the 2090s. Secondly, HAQI reveals larger and more persistent health burdens than the AQI. HAQI values consistently exceed AQI values and improve more slowly because HAQI aggregates excess risk from both PM_2.5_ and O_3_. For instance, under the SSP-RCP1-2.6 scenario, the AQI meets Target III one decade earlier than the HAQI, whereas under the SSP-RCP2-4.5 scenario, the AQI indicates moderate progress, yet the HAQI still suggests substantial health risk. This highlights the importance of complementing traditional concentration-based indices with health-based multipollutant metrics in air quality governance. Thirdly, the YRD is identified as a long-term multipollutant hotspot. Of the five city clusters, the YRD consistently exhibits the highest levels of PM_2.5_, O_3_, and HAQI. Particularly under the SSP-RCP3-7.0 scenario, the YRD's HAQI value is expected to reach approximately 140 by 2090. City-level analysis indicates that, under SSP-RCP1-2.6, only around 60% of YRD cities will achieve an HAQI of ≤ 50 by the 2080s. Under SSP-RCP2-4.5 and SSP-RCP3-7.0, however, no city will attain this “good” category, with all remaining in the “unhealthy” range.

Overall, these findings demonstrate that aligning China's development with a pathway similar to SSP-RCP1-2.6 would provide significant co-benefits for the climate mitigation and health protection by reducing PM_2.5_ and O_3_ levels. In this development pathways, it would lead to lower AQI and HAQI values, and help many regions to achieve WHO Target III. Conversely, weaker or delayed controls would make rapidly developing regions such as the YRD faced high-risk conditions. Therefore, our results support shifting the focus from simply “meeting concentration standards” to “ensuring health protection.” And using HAQI system and region-specific strategies for controlling multiple pollutants to jointly advance air-quality management and climate-mitigation goals.

## Data Availability

The original contributions presented in the study are included in the article/Supplementary material, further inquiries can be directed to the corresponding author.
