# Supplementary material for: Toward healthy air quality under CMIP6 scenarios: insights into the compliance with the new WHO guidelines in China
Source: Front Public Health. 2026 Mar 16;14:1803304. doi: 10.3389/fpubh.2026.1803304 (PMC13033637; doi:10.3389/fpubh.2026.1803304)
Supplement: Supplementary file 1 [file Data_Sheet_1.docx]

Supplementary Material for

**Towards Healthy Air Quality under CMIP6 Scenarios: Insights into the Compliance with the New WHO Guidelines in China**

**Ziqi Jia**^1^**, Yue Yuan**^2,3^**, Xurong Wang**^4^**, Fuzhen Shen**^5^**^*^**

^1^China Industrial Culture Research Center, Nanjing University of Science and Technology, Nanjing 210094, Jiangsu, China

^2^Key Laboratory of Meteorological Disaster Prevention and Mitigation, Shandong Provincial Meteorological Bureau, Jinan 250031, China

^3^Jining Meteorological Bureau, Jining 272000, China

^4^Institute of Climate and Energy System-Troposphere (ICE-3), Forschungszentrum Jülich GmbH, 52425 Jülich, Germany

^5^Institute of Climate and Energy System-Stratosphere (ICE-4), Forschungszentrum Jülich GmbH, 52425 Jülich, Germany

*** Correspondence:**

Fuzhen Shen

Email: [f.shen@fz-juelich.de](mailto:f.shen@fz-juelich.de)

**Supplementary Material list:**

**Supplementary Tables: Table. S1**

**Supplementary Figures: Fig. S1-S3**

**Table. S1** Bias metrics of bias-based CMIP6 of PM_2.5_ and O_3_ relative to CAMS

| Pollutant | NMB (%) | NME (%) | Pearson R |
| --- | --- | --- | --- |
| PM_2.5_ | -1.8 | 26 | 0.35 |
| O_3_ | -1.7 | 12 | 0.70 |

**Fig. S1.** Daily time series comparison between CAMS (green line), CESM2-WACCM (red line) and bias-corrected CESM2-WACCM (black line) for PM_2.5_ (top panel) and O_3_ (bottom panel) from 2003-2014.

**Fig. S2.** The bar plot of decadal mean PM_2.5_ from 2015-2090 under three different scenarios (left: SSP-RCP1.26, medium: SSP-RCP2.45, right: SSP-RCP3.70) in China, NCP, YRD, PRD, FWP and SCB respectively.

**Fig. S3.** The bar plot of decadal mean O3 from 2015-2090 under three different scenarios (left: SSP-RCP1.26, medium: SSP-RCP2.45, right: SSP-RCP3.70) in China, NCP, YRD, PRD, FWP and SCB respectively.
